# Supplementary figures and images for: Effects of Climate and Land Use on the Population Dynamics of the Bank Vole (Clethrionomys glareolus) in the Southernmost Part of Its Range
Source: Animals (Basel). 2025 Mar 14;15(6):839. doi: 10.3390/ani15060839 (PMC11939536; doi:10.3390/ani15060839)

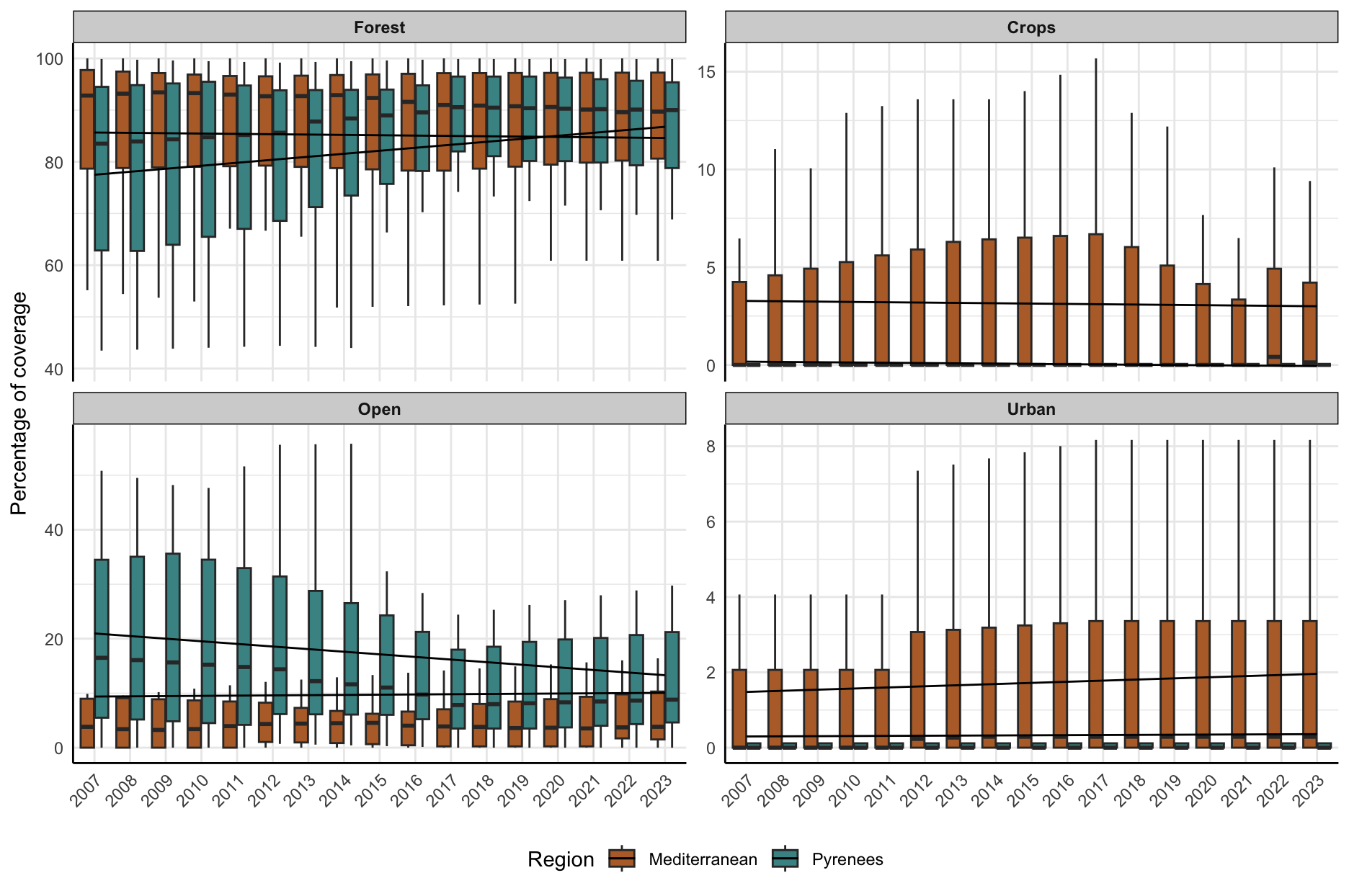

Supplement: Supplementary file 1 [file animals-15-00839-s001.zip › BankVole_FigureS2_DeLaHuertaSchliemann_et_al.png]

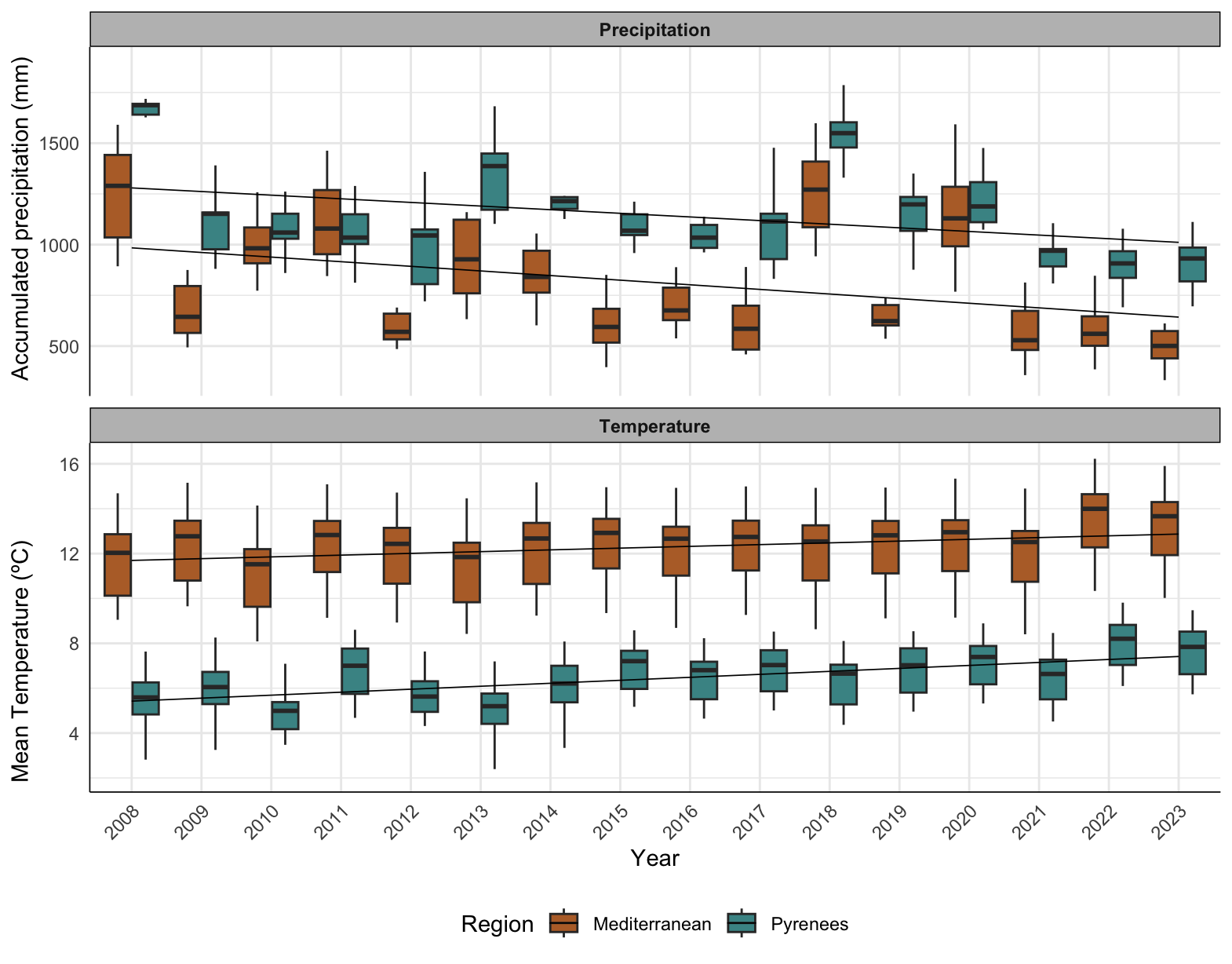

Supplement: Supplementary file 1 [file animals-15-00839-s001.zip › BankVole_FigureS1_DeLaHuertaSchliemann_et_al.png]
